# Holmium Laser Enucleation of the Prostate and Iatrogenic Arteriovenous Fistula Treated by Superselective Arterial Embolization

**DOI:** 10.1155/2016/4918081

**Published:** 2016-02-28

**Authors:** Anastasios D. Asimakopoulos, Lorenzo Dutto, Paolo Preziosi, Enrico Spera, Francesco Micali, Andrea De Carolis, Beniamino Iorio

**Affiliations:** ^1^Department of Experimental Medicine and Surgery, UOC of Urology, University of Rome Tor Vergata, Policlinico Casilino, Via Casilina 1049, 00169 Rome, Italy; ^2^Department of Urology, Prostatazentrum Nordwest, Möllenweg 22, 48599 Gronau, Germany; ^3^Department of Radiology, Policlinico Casilino, Via Casilina 1049, 00169 Rome, Italy; ^4^Department of Urology, Policlinico Casilino, Via Casilina 1049, 00169 Rome, Italy

## Abstract

Iatrogenic pelvic pseudoaneurysm with concomitant arteriovenous fistula has been described as a rare and challenging complication, which may occur during transurethral resection of the prostate. We provide the first report of this complication after holmium laser enucleation of the prostate for the treatment of benign prostatic hyperplasia. The attempt to control the bleeding by conversion to open surgery and placement of haemostatic stitches into the prostatic fossa failed. Angiography with superselective arterial embolization proved to be a modern, quick, safe, and efficient treatment of this uncommon complication.

## 1. Introduction

Iatrogenic, posttraumatic pelvic pseudoaneurysm and arteriovenous fistula (AVF) is a rare but described complication of pelvic surgery [1]. Endovascular techniques are increasingly used to control bleeding and occlude the AVF [1–3]. We report the first case of inferior vesical artery pseudoaneurysm and accompanying AVF secondary to holmium laser enucleation of the prostate. An open conversion failed to treat the bleeding, which was then successfully controlled by transarterial coil embolization.

## 2. Case Report

Transurethral holmium laser enucleation of the prostate (HoLEP) was carried out on a 62-year-old male patient with a history of benign prostatic hyperplasia. During the dissection of the right anterolateral surface of the adenoma, a large venous lumen was incised and pulsatile bleeding occurred. All the efforts of endoscopic control of the bleeding (using both bipolar and monopolar coagulation) were unsuccessful. Thus, an open conversion was performed. An infraumbilical midline laparotomy was used to gain access to the cavum Retzii and a midline longitudinal cystotomy was performed to access the bladder lumen. A circular incision was made on the vesical mucosa overlying the prostate lobes in the vicinity of the bladder neck area, which was deepened until the residual prostate adenoma was identified and digitally enucleated as for a classic suprapubic adenomectomy. Several haemostatic stitches were placed into the prostatic fossa. The catheter balloon was inflated with 60 cc and placed into the prostatic fossa to try to obtain haemostasis by local compression. An apparently acceptable haemostatic control had been achieved, so the surgery was concluded with the insertion of a pelvic drain and closure of the surgical access.

**Figure 1:**
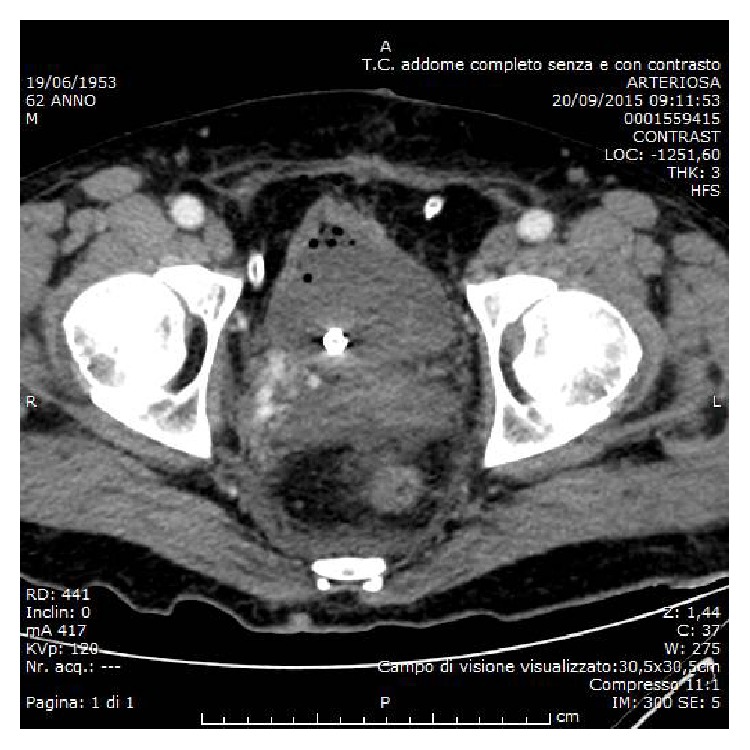
Contrast-enhanced CT scan, angiographic phase: abnormal extravasation of contrast medium in the soft tissue near bladder wall.

**Figure 2:**
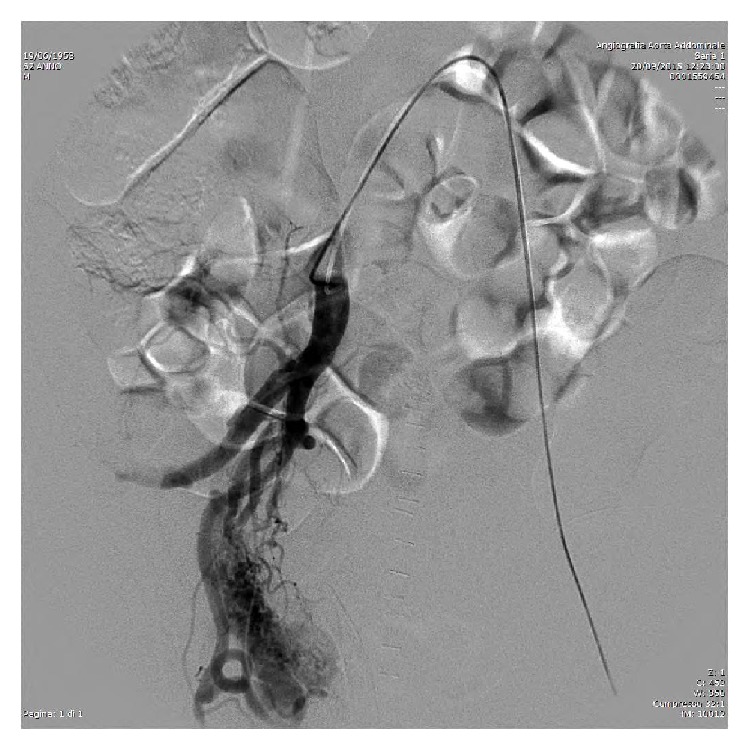
Angiography: before embolization, angiography demonstrates a contrast medium collection from vesical branches of the hypogastric artery and the bed of an arteriovenous fistula with early filling of the vein.

**Figure 3:**
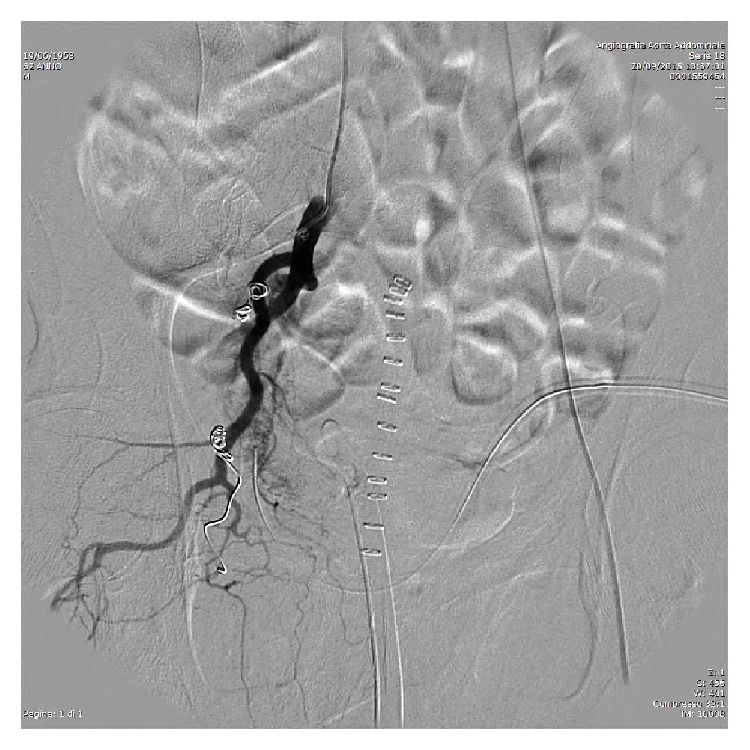
Angiography following embolization with metallic minicoils: occlusion of arterial branches and closure of the AV fistula without extravasation of contrast medium.

In the postoperative setting a progressive drop in haemoglobin required the transfusion of 6 units of blood. A contrast-enhanced CT scan of the abdomen and pelvis was carried out and revealed the presence of a 3 cm right pelvic pseudoaneurysm (Figure 1). The patient was promptly referred to the interventional radiology unit for further investigation. After selective catheterization of the left femoral artery, the injection of the right internal iliac artery was obtained. This confirmed the presence of a pseudoaneurysm with venous filling during the early arterial phase, suggesting an AVF (Figure 2) in addition to extravasation of the contrast medium. Following the selective catheterization of each single branch of the vesical, vesicoprostatic and obturator arteries, using a coaxial 2,7 French catheter (Progreat, Terumo), embolization was carried out by using minicoils 0,18 with diameter between 3 and 9 mm (Boston Scientific). After the embolization, control angiograms showed an almost complete occlusion of the feeding branches and no persistence of the AVF (Figure 3). Hematuria was totally resolved after the scleroembolization and the patient was dismissed two days later.

## 3. Discussion

Internal iliac artery pseudoaneurysm with concomitant AVF is a rare but potentially dramatic complication of the transurethral surgery for prostatic hyperplasia. Already described in the setting of laparoscopic radical prostatectomy [3–5] and transurethral resection of the prostate [2, 6], this is the first report of this complication in the field of HoLEP.

Pelvic pseudoaneurysms with concomitant, symptomatic AVF represent complex diagnostic and therapeutic challenges. During transurethral surgical treatment of the prostate, they should be suspected in case of excessive bleeding that cannot be controlled by standard endourologic and open techniques. The precise localization of fistulas, especially when they affect the branches of the internal iliac artery, may be challenging. The arterial phase of the contrast-enhanced CT scan represents a first step for an early diagnosis [7], while the selective angiography confirms the diagnosis and provides the means for a thorough control of the bleeding. If a clinically relevant residual flow and persistent arterial perfusion of the pseudoaneurysm/AVF are identified, selective surgical resection/ligation (open or laparoscopic) may be further adopted to achieve a complete closure of the arteriovenous communication.

In some cases such pseudoaneurysms may undergo spontaneous thrombosis [8], but in our case the concomitant presence of AVF resulted in abnormal hematuria requiring definitive treatment. The attempt to stop the bleeding through a transvesical access was unsuccessful, since the feeding artery was located outside the vesicoprostatic lumen. Consequently it could not be controlled with the hemostatic stitches, which are traditionally placed into the prostatic fossa during adenomectomy. Thus, a superselective coil embolization with a more cranial control of the bleeding is to be preferred in case an AVF is confirmed. More than one procedure may be required in case of residual or recurrent symptoms; additional surgical interventions subsequent to the embolization therapy may be also adopted [9]. However, complete, one-stage therapy is preferable due to surgical difficulties following failed or incomplete radiological or surgical attempts [10].

## 4. Conclusions

We report the first case of pseudoaneurysm with concomitant AVF occurring during HoLEP. While the classic surgical treatment may be complicated/unsuccessful due to the location of these lesions, endovascular techniques, implying superselective arterial embolization, represent a modern, quick, safe, and efficient treatment.
